# Nutritional composition and consumer acceptance of tomato paste fortified with palm weevil larvae (*Rhynchophorus phoenicis Fabricius)* in the Ashanti region, Ghana

**DOI:** 10.1002/fsn3.3418

**Published:** 2023-07-17

**Authors:** Loloah Chamoun, Salwa Karboune, Herman E. Lutterodt, Hugo Melgar‐Quinonez

**Affiliations:** ^1^ School of Human Nutrition McGill University; ^2^ Department of Food Sciences and Agriculture McGill University; ^3^ Department of Food Sciences and Technology Kwame Nkrumah University of Science and Technology

**Keywords:** acceptance, edible insects, fortification, sensory evaluation

## Abstract

Edible insects, such as palm weevil larvae, have been promoted as an alternative source of nutrients in developing countries for their nutritional benefits, cost‐effective rearing, and yearly availability. Unfortunately, consumer acceptance remains a barrier to their utilization. A supplemental palm weevil larvae and tomato paste were developed as part of efforts to understand whether incorporating edible insects into staple foods could help overcome this barrier. Palm weevil larvae flour and tomato paste were mixed in three formulations that had 8, 15, and 30% of palm weevil larvae flour. Samples were subjected to proximate and mineral content analyses and sensory evaluation. Among the blends, tomato paste containing 30% palm weevil larvae had the highest protein, fat, and total solids content as compared to unfortified tomato paste. Iron and zinc levels also increased with increasing levels of palm weevil larvae flour. Carbohydrate and crude fiber concentrations of the samples, however, decreased with increasing fortification levels. The overall acceptance and willingness to purchase fortified tomato paste as determined by sensory evaluation was high for all samples and increased with increasing knowledge about palm weevil larvae's nutritional benefits. Overall acceptance and willingness to purchase fortified tomato paste were significantly dependent on the samples' color and consumers' overall liking of the products. Tomato paste fortified with palm weevil larvae can provide a complementary source of iron for Ghanaians.

## Graphical abstract

This paper aimed to explore Ghanaian consumers' acceptance of varying fortification levels of palm weevil larvae–tomato paste and to investigate the main determinants for their willingness to pay for the products. This study found that the addition of palm weevil larvae improved the nutritional composition of tomato paste specifically in terms of its protein, fat, and zinc contents. The overall acceptance and willingness to purchase fortified tomato paste as determined by sensory evaluation using women of reproductive age were high for all samples and increased with increasing knowledge about palm weevil larvae's nutritional benefits.

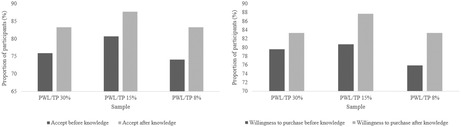

## 1 INTRODUCTION

Iron deficiency anemia remains a globally prevalent threat to women's health. In 2019, around 30% of women of reproductive age (WRA) were estimated to be anemic worldwide (Ritchie & Roser, 2017). Moreover, in Ghana, anemia affected over 35% of WRA in 2019 and 40% of the cases were attributed to iron deficiency (Safiri et al., 2021).

Various strategies have been promoted as effective ways of reducing iron deficiency anemia at population level including food fortification and iron supplementation. In Ghana, however, their implementation has not proven successful due to inadequate and inconsistent fortification across the country, inequitable distribution of supplements, and/or low adherence to supplementation (Appiah et al., 2020; Nasir et al., 2020; Osendarp et al., 2018). To overcome such challenges, food‐to‐food fortification is suggested as a complementary strategy that utilizes locally found underutilized animal‐ or plant‐based micronutrient‐rich sources to fortify nutritious, affordable, locally available, accessible, and acceptable food vehicles, commonly consumed by the population (Chadare et al., 2019). Evidence on the effectiveness of food‐to‐food fortification has been reported in the literature, particularly for increasing iron content and bioavailability (Kruger et al., 2020). A recent review assessed the impact of fortifying starchy staple foods with vegetables, fruits, and animal products at levels varying between 10% and 50% on the iron content and in vitro iron bioavailability (Kruger et al., 2020). Results from this review indicated an average increase of 50% in the fortified foods' iron content, as well as a significant increase in the iron's in vitro bioavailability (Kruger et al., 2020). Of all the studies assessed, one was of particular interest to this research as it evaluated the impact of adding mopane worms to fermented cereals for their iron content and bioavailability (Gabaza et al., 2018). Results from this study indicated a significant increase in most of the selected cereals' iron content but no significant change in their iron bioavailability.

Edible insects are considered important food sources to improve the iron status of resource‐limited populations owing to their nutritional value and cost‐effective farming. Edible insects are rich sources of complete animal protein and contain essential vitamins and minerals (such as iron and zinc) in quantities comparable to those of beef. Edible insects' nutritional value varies with insect's diet, sex, species, growth environment, and developmental stage (van Huis, 2021). Edible insects' protein content ranges from 35% to 60% (dry weight), their iron content ranges from 4 to 62 mg per 100 g of dry matter, and their zinc content ranges from 9 to 27 mg per 100 g of dry matter (Gorbunova & Zakharov, 2021; Latunde‐Dada et al., 2016; Mwangi et al., 2018). Insect consumption, or entomophagy, is practiced by over 2 billion people in 113 countries in Africa, Asia, and Latin America (van Huis, 2021). In Ghana, the most commonly consumed insects are termites, shea tree caterpillars, grasshoppers, locusts, field crickets, and palm weevil larvae (*Rhynchophorus phoenicis Fabricius*) (Anankware et al., 2016). Palm weevil larvae, locally called *akokono*, are available all year long in palm‐growing communities, and are particularly abundant during the rainy season from May to October (Anankware et al., 2016).

Despite the numerous benefits of edible insects, consumer acceptance remains a barrier to their utilization due to the disgust factor associated with their consumption (Imathiu, 2020). As such, to overcome this obstacle, incorporating edible insects into staple foods could promote their utilization as a food source and help reduce micronutrient deficiencies and malnutrition. For a successful fortification, the selection of the appropriate food vehicle, fortificant, and level of fortification is crucial. According to the Food and Agriculture Organization and the World Health Organization (Allen, De Benoist, Dary, Hurrell, & Organization, 2006; Chadare et al., 2019), the food vehicle should be consumed frequently and in adequate quantities by the majority of the population, accessible financially and physically, and centrally processed. As for the fortificants, underutilized plant or animal species that are nutrient‐rich and require little agricultural inputs are preferred. Finally, the fortification level should vary between 1% and 50% in such a way that the fortificant enhances the nutritional quality of the food vehicle without compromising its organoleptic properties or its acceptability by consumers.

Tomato paste was considered a potential food vehicle to be fortified with palm weevil larvae. Fresh and canned tomatoes (such as tomato paste) are a staple food in Ghanaians' diets and are widely consumed in Ghana (Ministry of Food and Agriculture (MoFA), 2020). According to a study conducted by Aggey et al. (2007), tomato paste is used by at least 7 in 10 Ghanaian households in preparing their meals during lean tomato season. Tomato paste constitutes the base of several Ghanaian stews and dishes. Tomatoes and its derivatives are generally good sources of essential nutrients, although low in iron (Sainju et al., 2003), and appear to have low levels of antinutrients including phytates, glycosides, saponins, and tannins (Oyetayo & Ibitoye, 2012).

To evaluate the feasibility, effectiveness, and sustainability of this strategy, it is necessary to assess consumers' acceptability of the developed product and evaluate the nutritional changes imparted by the addition of *akokono* flour. To the best of our knowledge, no study has looked at consumer acceptance of palm weevil larvae‐based tomato paste. Thus, the objective of this study was to explore Ghanaian consumers' acceptance of varying fortification levels of palm weevil larvae–tomato paste and to investigate the main determinants for their willingness to pay (WTP) for the products.

## 2 MATERIALS AND METHODS

Using the Association of Official Analytical Chemists (AOAC) methods (Association of Official Analytical Chemists, 1995), we examined the nutritional composition of *akokono*‐based tomato paste. A sample of 88 untrained panelists consisting of community men and women conducted a sensory evaluation of three tomato paste samples with varying concentrations of *akokono* flour. Finally, these participants evaluated their acceptance and willingness to purchase the tested samples.

### 2.1 Raw materials

Samples of tomato paste with varying palm weevil larvae compositions were prepared. Full‐fat palm weevil larvae flour was provided by Legendary Foods' farm site in Kumasi, Ghana. All the other ingredients including the tomato paste were purchased from a local market in Kumasi, Ghana. The sensory evaluation was carried out in selected communities in Kumasi Ghana, while the analyses were performed in laboratories of the Department of Food Science at McGill University, Canada. All reagents used for the proximate and mineral analyses were obtained from Sigma Aldrich Co. (USA).

### 2.2 Preparation of palm weevil larvae‐fortified tomato paste

The palm weevil larvae‐fortified tomato paste was prepared by mixing 100 g of tomato paste with varying weights of *akokono* flour (8, 15, and 30 g) and heating it for 5 to 10 minutes at 70 °C until the appropriate texture was reached.

### 2.3 Proximate analysis

The proximate analysis was carried out according to AOAC (Association of Official Analytical Chemists, 1995) and done in triplicate.

#### 2.3.1 Determination of moisture content

Moisture content of the tomato paste was determined according to the official methods of analysis (AOAC 927.05) (Association of Official Analytical Chemists, 1995). Samples were weighed and placed into dried and weighed moisture dishes before being dried in a vacuum oven at 70 °C overnight, after which the dishes were placed in a desiccator to cool to room temperature and until a constant weight was obtained. The moisture content was expressed as a percentage of the average weight loss after drying the samples based on the following formula: $\frac{W2‐W3}{W2‐W1}*100$ where W1 is the weight of the dish, W2 is the weight of the dish and the initial wet sample, and W3 is the weight of the dish with the dried sample.

#### 2.3.2 Determination of ash content

Ash is defined as the inorganic residue left after the food's organic residue has been burnt. Ash content was determined the according to the official methods of analysis (AOAC 942.05) (Association of Official Analytical Chemists, 1995). *Akokono*‐based tomato paste samples were weighed (1 g each) and transferred into dry and weighed crucibles. Samples were incinerated for 2 h at 550°C in a muffle furnace. Samples were then allowed to cool to room temperature before being placed in a desiccator and weighed afterward. The difference in weight was expressed as the ash percentage in the tomato paste: ${\frac{W2‐W3}{W1-W3}}^{*}100$, where W1 is the weight of the crucible and raw sample, W2 is the weight of the crucible and dried sample, and W3 is the weight of the crucible.

#### 2.3.3 Determination of crude protein

The Dumas method (Association of Official Analytical Chemists, 1995) was used to determine the crude protein content of the *akokono* tomato paste samples. Samples were freeze‐dried and then weighed (8–12 mg) into metal caps (which had been blanked beforehand). The amount of nitrogen was then quantified using a universal detector, NC Soil Analyzer, Flash 1112 Series EA (Thermo Finnigan). The crude protein content of samples is calculated by multiplying the percentage of nitrogen by 6.25.

#### 2.3.4 Determination of crude fat

The Soxhlet extraction method (Association of Official Analytical Chemists, 1995) was used for crude fat determination. Extraction cups were weighed before adding 50 mL of petroleum ether (solvent). Dried samples from the moisture determination were weighed (2 to 3 g each) and transferred into extraction thimbles. The thimble and the sample were then placed in a Soxhlet extractor (SER 148, Solvent Extraction VELP Scientifica) for about an hour and a half. Once the extraction was completed, the extraction cups were weighed, and the crude fat content was calculated according to the following formula:

$${\frac{\text{Weight of extraction}\;\mathrm{cup}\;\text{with}\;\mathrm{fat}‐\text{Weight of extraction}\;\mathrm{cup}}{\text{Weight of sample}}}^{*}100$$

#### 2.3.5 Determination of carbohydrates

Total carbohydrates were determined by difference according to the following formula:

$$100‐\left(\%\text{crude}\;\mathrm{fat}+\%\mathrm{ash}+\%\text{crude protein}\right)=\text{carbohydrate content}$$

#### 2.3.6 Determination of dietary fiber

Dietary fiber was determined through a combination of enzymatic and gravimetric methods. Dietary fiber content was determined using the total dietary fiber assay kit (Sigma‐Aldrich Co., USA) based on the AOAC 985.26 method. Defatted samples were weighed into beakers (0.5 g) and gelatinized using α‐amylase. Then, they were enzymatically digested with 5 mg of protease and 0.1 mL of amyloglucosidase to remove the protein and starch present in the sample. Ethanol (200 mL) was then added to precipitate the soluble dietary fiber. The residue was then filtered and washed with ethanol and acetone. After drying in a vacuum oven at 70°C, the residue was weighed. Half of the samples were analyzed for protein and the others were ashed. Total dietary fiber is the weight of the residue minus the weight of the protein and ash.

### 2.4 Mineral analysis

Mineral analysis was carried out in order to determine the content of iron and zinc using inductively coupled plasma mass spectrometry (ICP‐MS) (Jajda et al., 2015). Samples of 0.5 g each were weighed into 50‐mL pre‐cleaned Falcon tubes to which were added 6 mL of trace‐metal‐grade nitric acid. The samples were heated in a water bath at 90°C for 2 hours and then allowed to cool at room temperature. Hydrogen peroxide (3 mL) was added to these samples which were then heated in a water bath at 90°C for an additional hour until particulates were not visible. Solutions were diluted to 2% nitric acid and analyzed using ICP‐MS. All mineral analyses were performed in duplicate.

### 2.5 Sensory evaluation of tomato paste (hedonic tasting)

A consumer acceptability study was conducted among 88 participants (panelists) from five selected communities in Kumasi for all three tomato paste formulations. Each panelist evaluated a sample set of three random samples which were each assigned a three‐digit blinding code. Kilcast's recommendations for sample presentation were respected (Kilcast, 2010). Saltine crackers were used as palate cleansers. Panelists were asked to rate the samples on the following parameters: color, taste, texture and consistency, mouthfeel, and overall liking using a 5‐point Likert scale. Panelists were also questioned about their overall acceptance of the products and their willingness to purchase them. They were then briefed on the nutritional benefits of palm weevil larvae after which they were asked to answer the previous questions again. All participants were informed about the allergenic potential of insects and the ones with a known shellfish allergy were denied participation. Participants were informed that their participation was voluntary and were asked to sign a consent form.

### 2.6 Statistical analyses

Sensory evaluation data were analyzed using frequencies and proportions for categorical variables and McNemar's test to evaluate whether the proportions of customers accepting the products and willing to purchase them were significantly different before and after having been informed about the benefits of *akokono*. Analysis of variance (ANOVA) was performed to see if there was a significant difference in the overall liking of all samples at p < 0.05. Multivariate analysis of variance (MANOVA) was then performed to determine if the samples were significantly different considering all the sensory attributes simultaneously at p < 0.05. Binary logistic regression analyses were finally performed to identify the sensory attributes that were critical to the overall acceptance of the samples and respondents' purchase intent. ANOVA and Tukey's honestly significant difference test (p < 0.05) were used to detect significant differences among the nutrient contents of the different formulations.

## 3 RESULTS

### 3.1 Determination of nutrient composition

The nutrient composition of the tomato paste enriched with *akokono* is presented in Table 2 (dry basis), Table 3 (wet basis), and Table 4 (per 200 g of tomato paste). Tomato paste samples' total solids content ranged from 27.3% to 58.4%. Total solids increased with increasing *akokono* flour concentration. Results from the wet basis analysis showed that the tomato paste samples' protein content ranged from 4.59 to 13.6 g per 100 g of tomato paste (wet basis). An increase in the protein content of tomato paste was observed with increasing *akokono* concentration. The protein content of the 30:100 *akokono‐*fortified tomato paste sample was significantly higher than that of the unfortified tomato paste. As for tomato paste's ash content, it increased significantly with an increasing concentration of *akokono* flour, going from 1.88 to 3.09 g per 100 g of tomato paste (wet basis). Tomato paste's fat content increased significantly with the addition of *akokono,* increasing from 0.09 to 16.5 g per 100 g of tomato paste (wet basis). No specific trends were detected for fortified tomato paste's fiber and carbohydrate contents which varied between 1.13 and 2.45 g and 18.3 and 25.2 g, respectively, per 100 g of tomato paste (wet basis). Moreover, the addition of *akokono* significantly increased tomato paste's iron and zinc contents (wet basis). Tomato paste's iron content increased from 1.74 to 3.83 mg per 100 g of tomato paste (wet basis) and its zinc content increased from 0.16 to 1.99 mg per 100 g of tomato paste (wet basis) with increasing concentrations of *akokono* flour. As for *akokono* flour, it had a low moisture content (5.92%), high protein (23.9 g per 100 g wet basis) and fat (54.1 g per 100 g wet basis) contents, moderate carbohydrate (11.8 g per 100 g wet basis) and fiber (5.36 g per 100 g wet basis) contents, and good iron (3.74 mg per 100 g wet basis) and zinc contents (12.1 mg per 100 g wet basis).

The estimated amount of nutrients that would be consumed on a daily basis assuming a consumption of 200 g of tomato paste per day was presented in Table 4. Our findings showed that consuming 200 g of the 30:100 *akokono‐*fortified tomato paste would provide twice as much iron (7.66 mg), almost three times more protein (27.2 g), and 12 times more zinc (3.98 mg) than consuming the same amount of unfortified tomato paste.

A one‐way ANOVA and Tukey's honestly significant differences test were performed to see whether the addition of palm weevil larvae to tomato paste had a significant impact on tomato paste's nutrient composition. Results from the ANOVA test revealed that samples had significantly different ash, moisture, fat, carbohydrate, fiber, iron, and zinc contents. Results from Tukey's honestly significant differences test comparing the significant difference in samples' means for each nutrient are presented in Tables 2, 3, and 4.

### 3.2 Sensory evaluation

#### 3.2.1 Characteristics of participants and purchasing frequency

**FIGURE 1:**
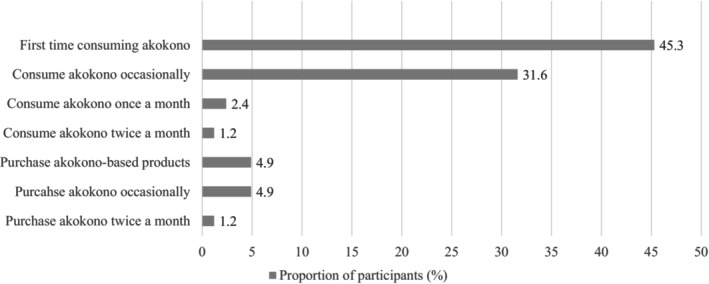
Akokono consumption and purchasing habits of respondents.

As seen in Table 1, most panelists were women (77.5%) and a third of them were aged between 25 and 34 years old (33%). Only a few participants had a university degree (19.3%), while almost half had completed secondary education (46.8%). Over 90% of participants were low‐income earners with 40% earning below 500 GHC per month (equivalent to 47 USD per month). When asked about their awareness of iron and its importance in their diets, only half of the participants reported being well‐informed on iron and recognizing its significance for their health and well‐being. Cross‐tabulations and Chi‐square tests were conducted between participants' socio‐demographic characteristics (age, sex, income level, and educational attainment) and their willingness to purchase *akokono*‐based tomato paste. No significant association was found. Almost half of the participants (45%) reported consuming *akokono* for the first time during this sensory evaluation, while 31.6% indicated being occasional consumers (less than once a month). Consuming *akokono*, however, was more popular than purchasing it as 31% of participants engaged in the former while 5% engaged in the latter, as seen in Figure 1. This implies that *akokono* is either being offered as a delicacy or is self‐harvested.

**TABLE 1:** Socio‐demographic characteristics of participants and iron knowledge.

	Proportion of participants (%)
**Gender**	
Women	81.8
Men	18.2
**Age group (years old)**	
18–24	24.3
25–34	35.6
35–49	17.0
50+	23.1
**Income level (Ghanaian Cedis/month)**	
Below 500	42.6
500–1500	53.8
1501–2000	1.20
2001–5000	2.40
**Educational attainment**	
No degree	14.6
Primary education	15.8
Secondary education	48.6
University degree	21.0
**Knowledge of iron**	
Yes	51.4
No	48.6
**Importance of iron**	
Very important	47.7
Slightly important	2.40
I do not know	49.8

#### 3.2.2 Likert scale scores for overall liking and sensory attributes

**FIGURE 2:**
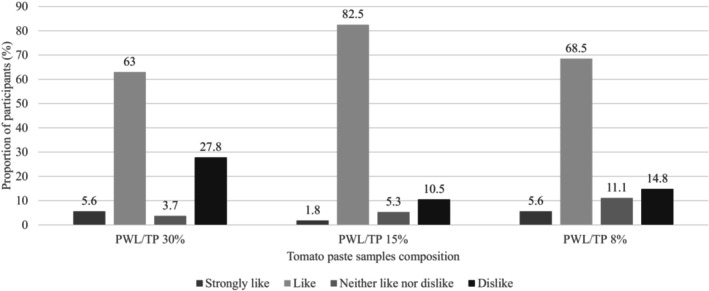
Overall liking of tomato paste samples and like‐to‐dislike ratio of samples.

The median Likert scale rating for all samples was 2, or “Like,” indicating that participants generally liked all samples. As seen in Figure 2, tomato paste sample [PWL/TP (palm weevil larvae/tomato paste), 15%] was the most preferred by over 80% of the participants and had the highest like‐to‐dislike ratio. On the contrary, sample [PWL/TP, 30%] was the least liked by participants. Results from the ANOVA test, as seen in Table 5, indicate that the difference in overall liking between all three samples is significant (F = 6.077, p < 0.05).

When looking at the sensory attributes evaluated for tomato paste, as seen in Annex 1‐4, we noticed that the color of sample [PWL/TP, 15%], the taste of sample [PWL/TP, 15%], the texture/consistency of sample [PWL/TP, 15%], and the mouthfeel of sample [PWL/TP, 15%] were the most liked. There was a significant difference in the color (F = 6.713, p < 0.05), taste (F = 7.476, p < 0.05), texture/consistency (F = 3.236, p < 0.05), and mouthfeel (F = 3.795, p < 0.05) ratings of all samples (see Table 7).

**FIGURE 3:**
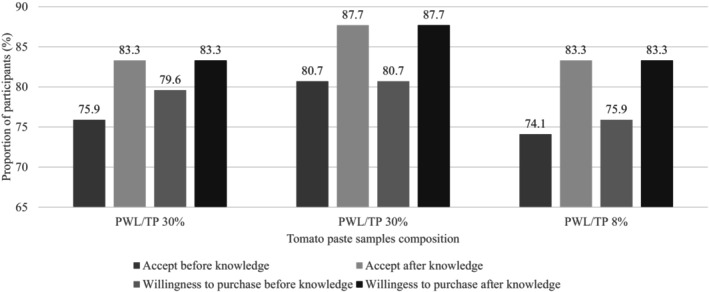
Acceptance and willingness to purchase *akokono* products before and after knowledge of benefits.

#### 3.2.3 Statistical interpretation of tomato paste fortified with *akokono* formulations

For the multivariate analysis of variance (MANOVA), Wilks lambda's F‐value greater than 1 and p‐value less than 0.05 indicate that all formulations were perceived by the panelists as different (as seen in Table 6). In addition, the total‐sample standardized canonical coefficient indicates the attribute that is more responsible for the differences. Our results from canonical 1 (Table 6) indicated that color and taste were the most responsible for the differences. Logistic regression was then performed with “overall acceptance” and “willingness to purchase” as the dependent variables and the sensory attributes as the independent variables. According to the results of logistic regression analysis, color and overall liking were found to be the most significant (α = 0.05) determinants for the overall acceptance of *akokono‐*fortified tomato paste fortified and consumers' willingness to purchase it.

#### 3.2.4 Influence of knowledge on acceptance and willingness to purchase

As seen in Figure 3 according to McNemar's test, there was a significant increase in respondents' willingness to purchase and accept all samples for consumption after they were informed about the potential nutritional benefits of consuming *akokono*. However, these differences were only significant overall, but not when looking at each sample separately.

## 4 DISCUSSION

This study presents the nutritional composition of tomato paste samples fortified with palm weevil larvae (*akokono*), an edible insect consumed in Ghana, and evaluates Ghanaian community members' acceptance and willingness to purchase the prepared product formulations.

### 4.1 Nutrient composition of *akokono‐*based tomato paste

The samples' macronutrient composition will be analyzed based on the dry‐basis values obtained (Table 2) in order to compare them to those found in the literature. The addition of *akokono* increased tomato paste's total solids content thus increasing its nutrient content. The addition of *akokono* significantly increased tomato paste's fat content, suggesting that it will contain higher amounts of fat‐soluble vitamins, essential fatty acids, and energy. The *akokono* flour's crude fat content (57.5% ± 0.35) was in agreement with values reported in the literature, which varied between 52.4% and 66.6% (Chinarak et al., 2020; Edijala et al., 2009; Ekpo & Onigbinde, 2005; Elemo et al., 2011; Omotoso & Adedire, 2007; Parker et al., 2017; Womeni et al., 2012). *Akokono*'s high‐fat content is seen to contribute to its acceptable taste and flavor profile when roasted; however, it may increase its susceptibility to storage deterioration via lipid oxidation (Ekpo & Onigbinde, 2005).

**TABLE 2:** Nutrient composition of tomato paste samples with varying palm weevil larvae flour concentrations (dry basis).

Samples ratio PWL:TP	Moisture content (%)	Ash content (% dry weight)	Protein content (% dry weight)	Fat content (% dry weight)	Carbohydrate content (% dry weight)	Fiber content (% dry weight)	Iron content (mg/100 g dry matter)	Zinc content (mg/100 g dry matter)
0:100	72.7 ± 0.07^a^	6.87 ± 0.10^a^	16.8 ± 0.14^a^	0.33 ± 0.21^a^	76.0 ± 0.71^a^	4.17 ± 0.20^a^	6.36 ± 0.09^a^	0.61 ± 0.28^a^
8:100	64.8 ± 0.49^b^	6.61 ± 0.05^a^	18.3 ± 0.05^b^	19.0 ± 0.35^b^	56.1 ± 0.77^b^	5.20 ± 0.57^b^	5.66 ± 0.47^b^	2.27 ± 0.19^a^
15:100	62.8 ± 0.49^c^	6.34 ± 0.39^a^	22.8 ± 0.21^c^	21.7 ± 0.14^c^	49.1 ± 0.19^c^	6.60 ± 0.19^b^	6.70 ± 0.14^a^	3.00 ± 0.35^b^
30:100	41.6 ± 0.21^d^	5.29 ± 0.21^b^	23.3 ± 0.42^c^	28.2 ± 0.18^d^	43.2 ± 0.15^d^	3.20 ± 0.03^c^	6.56 ± 0.11^a^	3.41 ± 0.06^b^
100:0	5.92 ± 0.06^e^	4.49 ± 0.07^b^	25.5 ± 0.28^d^	57.5 ± 0.35^e^	12.5 ± 0.37^e^	5.70 ± 0.007^b^	3.98 ± 0.18^c^	12.9 ± 0.63^c^

PWL: Palm weevil larvae flour.

TP: Tomato paste.

All values are expressed as mean ± SD. Sample means with different superscript letters in the same column are significantly different (p < 0.05).

**TABLE 3:** Nutrient composition of tomato paste samples with varying palm weevil larvae flour concentrations (wet basis).

Samples ratio PWL:TP	Moisture content (%)	Ash content (g/100 g wet basis)	Protein content (g/100 g wet basis)	Fat content (g/100 g wet basis)	Carbohydrate content (g/100 g wet basis)	Fiber content (g/100 g wet basis)	Iron content (mg/100 g wet basis)	Zinc content (g/100 g wet basis)
0:100	72.7 ± 0.07^a^	1.88 ± 0.10^a^	4.59 ± 0.14^a^	0.09 ± 0.21^a^	20.7 ± 0.71^a^	1.13 ± 0.20^a^	1.74 ± 0.09^a^	0.16 ± 0.28^a^
8:100	64.8 ± 0.49^b^	2.33 ± 0.05^b^	6.44 ± 0.05^ab^	6.68 ± 0.35^b^	19.7 ± 0.77^ab^	1.83 ± 0.57^b^	1.99 ± 0.47^b^	0.80 ± 0.19^b^
15:100	62.8 ± 0.49^c^	2.36 ± 0.39^c^	8.48 ± 0.2^ab^	8.07 ± 0.14^c^	18.3 ± 0.19^b^	2.45 ± 0.19^c^	2.49 ± 0.14^c^	1.11 ± 0.35^c^
30:100	41.6 ± 0.21^d^	3.09 ± 0.21^d^	13.6 ± 0.42^b^	16.5 ± 0.18^d^	25.2 ± 0.15^c^	1.87 ± 0.03^b^	3.83 ± 0.11^d^	1.99 ± 0.06^d^
100:0	5.92 ± 0.06^e^	4.22 ± 0.07^e^	23.9 ± 0.28^c^	54.1 ± 0.35^e^	11.8 ± 0.37^d^	5.36 ± 0.007^d^	3.74 ± 0.18^d^	12.1 ± 0.63^e^

PWL: Palm weevil larvae flour.

TP: Tomato paste.

All values are expressed as mean ± SD. Sample means with different superscript letters in the same column are significantly different (p < 0.05).

**TABLE 4:** Nutrient composition of tomato paste samples with varying palm weevil larvae flour concentrations (on consumed basis).

Samples ratio PWL:TP	Moisture content (%)	Ash content (g/200 g tomato paste)	Protein content (g/200 g tomato paste)	Fat content (g/200 g tomato paste)	Carbohydrate content (g/200 g tomato paste)	Fiber content (g/200 g tomato paste)	Iron content (g/200 g tomato paste)	Zinc content (g/200 g tomato paste)
0:100	72.7 ± 0.07^a^	3.75 ± 0.10^a^	9.17 ± 0.14^a^	0.18 ± 0.21^a^	41.5 ± 0.71^a^	2.28 ± 0.20^a^	3.47 ± 0.09^a^	0.33 ± 0.28^a^
8:100	64.8 ± 0.49^b^	4.65 ± 0.05^b^	12.8 ± 0.05^ab^	13.4 ± 0.35^b^	39.5 ± 0.77^ab^	3.66 ± 0.57^b^	3.99 ± 0.47^b^	1.60 ± 0.19^b^
15:100	62.8 ± 0.49^c^	4.72 ± 0.39^c^	16.9 ± 0.2^ab^	16.1 ± 0.14^c^	36.6 ± 0.19^b^	4.91 ± 0.19^c^	4.99 ± 0.14^c^	2.23 ± 0.35^c^
30:100	41.6 ± 0.21^d^	6.18 ± 0.21^d^	27.2 ± 0.42^b^	32.9 ± 0.18^d^	50.5 ± 0.15^c^	3.74 ± 0.03^b^	7.66 ± 0.11^d^	3.98 ± 0.06^d^
100:0	5.92 ± 0.06^e^	8.45 ± 0.07^e^	47.9 ± 0.28^c^	108 ± 0.35^e^	23.5 ± 0.37^d^	10.7 ± 0.007^d^	7.49 ± 0.18^d^	24.3 ± 0.63^e^

PWL: Palm weevil larvae flour.

TP: Tomato paste.

All values are expressed as mean ± SD. Sample means with different superscript letters in the same column are significantly different (p < 0.05).

**TABLE 5:** ANOVA for overall liking of tomato paste samples.

	Sum of squares	Df	Mean square	F	Sig.
Between groups	15.788	4	3.947	5.034	.001
Within groups	167.001	213	.784		
Total	182.789	217			

**TABLE 6:** Sensory analyses: sensory attributes critical to acceptance and purchase intent of tomato paste with varying concentrations of *akokono* flour.

MANOVA						
Statistic	Value	*F* value	Num DF		Den DF	p‐value
Wilks' lambda	0.794	3.04	25		1186.5	<.0001
Pillai's trace	0.216	2.92	25		1615	<.0001
Hotelling–Lawley trace	0.246	3.13	25		773.66	<.0001
Roy's greatest root	0.178	11.53	5		323	<.0001

Total‐sample standardized canonical coefficients						
Variable	**Canonical 1**	**Canonical 2**				
Color	0.625	0.672				
Taste	0.964	−1.58				
Texture/ Consistency	−0.482	−0.996				
Mouthfeel	−0.132	0.994				
Overall Liking	0.223	1.015				

Logistic regression analysis for acceptance*						
Variable		**Estimate**	**χ** ^**2**^	**p‐value**	**Odd's ratio**	
Color		−0.901	7.22	.0072	0.406	
Taste		−0.367	0.465	.499	0.693	
Texture/consistency		−0.00688	0.0002	.989	0.993	
Mouthfeel		−0.0960	0.0243	.876	0.908	
Overall liking		−2.33	14.06	.0002	0.097	

Logistic regression analysis for willingness to purchase*						
Variable		**Estimate**	**χ** ^**2**^	**p‐value**	**Odd's ratio**	
Color		−0.533	3.99	.0458	0.587	
Taste		−0.549	1.72	.189	0.578	
Texture/consistency		−0.135	0.089	.766	0.873	
Mouthfeel		−0.296	0.348	.555	0.744	
Overall liking		−1.246	7.68	.0056	0.288	
*Significance, α = 0.05						

**TABLE 7:** ANOVA for sensory attributes of tomato paste samples.

		Sum of squares	Df	Mean square	F	Sig.
Color	Between Groups	14.567	4	3.642	6.713	.000
	Within Groups	115.547	213	.542		
	Total	130.115	217			
Taste	Between Groups	24.576	4	6.144	7.476	.000
	Within Groups	175.043	213	.822		
	Total	199.619	217			
Texture/ Consistency	Between Groups	9.693	4	2.423	3.236	.013
	Within Groups	159.518	213	.749		
	Total	169.211	217			
Mouthfeel	Between Groups	11.591	4	2.898	3.795	.005
	Within Groups	162.629	213	.764		
	Total	174.220	217			

The addition of *akokono* increased tomato paste's protein content and complemented efficiently its amino acid composition. Our proximate analyses showed that *akokono* and tomato paste's protein contents (25.5% and 16.8% dry weight basis, respectively) corroborated the values reported in the literature stating that *akokono*'s protein content varied between 19.50% and 69.78% (dry weight basis) (Rumpold & Schlüter, 2013), and tomato paste's protein content ranged between 10.50% and 25.03% (Ali et al., 2021). According to several studies, palm weevil larva contains almost all of the essential amino acids in adequate quantities except for tryptophan (Chinarak et al., 2020; Ekpo & Onigbinde, 2005; Elemo et al., 2011; Womeni et al., 2012). Of particular interest are its high levels of leucine, lysine, and threonine, which are limiting amino acids in grains and cereals. Tomato paste contains on average 2.80 mg of leucine, 2.45 mg of lysine, and 1.37 mg of threonine per 100 g of protein (Ali et al., 2021), which is almost 15 times less than what is found in *akokono*. *Akokono* are comprised of 47 to 59 mg of leucine, 42 to 64 mg of lysine, and 29 to 31 mg of threonine per 100 g of protein (Rumpold & Schlüter, 2013).

The dry weight basis fiber content remained relatively stable with the addition of palm weevil larvae. This was expected as both tomato paste and palm weevil larvae have similar fiber contents. Canned tomato paste's fiber content has been reported in the literature as varying between 4.97% and 6.61% (dry weight basis) (Abdullahi et al., 2016) which aligns with the value we found (4.17% ± 0.20), while *akokono*'s fiber content has been reported as varying between 2.58% and 22.9% (dry weight basis) (Rumpold & Schlüter, 2013), which compares to our *akokono*'s fiber results of 5.70% ± 0.007 (dry weight).

The carbohydrate composition (dry basis) of the fortified tomato paste decreased with increasing concentrations of *akokono* flour. This was expected as *akokono's* carbohydrate content is lower than that of tomato paste. Our *akokono's* carbohydrate content (dry weight basis) (12.51% ± 0.37) corroborated the ranges found in the literature which varied from 4.21% to 48.50% (dry weight basis) (Ekpo & Onigbinde, 2005; Rumpold & Schlüter, 2013; Womeni et al., 2012).

As for the iron and zinc contents, their wet‐basis values were analyzed as they were more indicative of the amount that would be theoretically consumed. The addition of *akokono* increased significantly tomato paste's zinc and iron contents (wet basis) with increasing *akokono* concentrations. Moreover, a daily consumption of 200 g of 30:100 *akokono‐*fortified tomato paste would theoretically provide women with twice as much iron (7.66 mg of iron) than the unfortified tomato paste (3.47 mg of iron) would. This finding is important as it suggests that fortifying tomato paste with 30% (or more) *akokono* could significantly improve women's daily consumption of iron.

### 4.2 Sensory evaluation of samples fortified with *akokono* flour and factors influencing consumer acceptability and willingness to purchase

Our results showed no typical consumer profile for *akokono* as participants from different genders, age groups, income levels, and educational attainments were all interested in *akokono* and expressed willingness to purchase *akokono*‐based products.

The logistic regression analyses showed that the overall acceptance of the tomato paste samples and consumers' willingness to purchase them were significantly dependent on the samples' color and consumers' overall liking of the products (p < 0.05). Our findings corroborate with Smarzyński et al. (2019) and their colleagues who also identified color as a determining factor in consumers' acceptance of a pork pâté fortified with cricket powder (Smarzyński et al., 2019). Visual cues like color are essential determinants of a food product's acceptance since they are indicators of a product's freshness (Arce‐Lopera et al., 2015; Grunert, 1997). As a result, if a product's color is unacceptable, flavor and texture are unlikely to be properly judged. When Keneko and colleagues (2002) evaluated consumer acceptability of color in tomato‐processed products, they determined that a peak color acceptance existed for all tomato products, stating that those were undesirable if they were too red or too brown (Claybon & Barringer, 2002). For tomato paste specifically, they found that consumers preferred it to be closer to the “red” end of the spectrum, indicating that consumers wanted a fresher product, rather than a more processed one, which would have been associated with a browner product (Claybon & Barringer, 2002). Taste and texture also motivated women's overall acceptance and willingness to purchase the *akokono‐*based tomato paste, if sold on the market. The sensory evaluation was performed on women, majorly (over 80%), as they are responsible for food purchasing and preparation in the household. When asked to justify their liking and willingness to purchase, women mentioned they were motivated by the fact that the tomato paste's taste and color remained appealing despite adding *akokono*. Some specified they would only buy *akokono* if it were added to tomato paste, as they would know how to cook it and could seamlessly incorporate it into theirs and their children's diets. The use of palm weevil larvae in a known and commonly consumed food product such as tomato paste seemed to have increased familiarity with *akokono* and reduced the taboo associated with entomophagy. Familiarity, which is achieved by integrating insects into already well‐known products and combining favorite dishes with insects, has been reported as essential in increasing acceptance of edible insects and decreasing neophobia among consumers (Wendin & Nyberg, 2021).

Most women preferred the tomato paste sample with the second highest concentration of *akokono* and indicated that they would use it in stews to diversify their diets and make them more nutritious. Health and nutrition were factors that motivated women's acceptance of *akokono*. A few other studies reported that nutrition was a major motive for insect consumption in developing countries (M. A. Ayieko & Oriaro, 2008; Manditsera et al., 2018; Obopile & Seeletso, 2013). Although respondents did not particularly know the insects' exact nutritional compositions, they perceived them as being highly nutritious foods that are rich in health‐promoting components (Manditsera et al., 2018). When women were informed about *akokono*'s health benefits, including its potential to improve their iron status, their acceptance and willingness to purchase the products increased, although not significantly. We found, indeed, no significant association between participants' willingness to purchase *akokono*‐based tomato paste and their iron knowledge. Out of the participants willing to purchase it, 50% reported being knowledgeable about iron. The link between nutrition knowledge and dietary habits, however, has been established in African countries as listed in Worsley's article (Worsley, 2002), including in Ghana (Rose Omari, 2017) and Ethiopia (Melesse & van den Berg, 2021). A study conducted in Ethiopia highlighted that nutrition knowledge was positively related to healthy attitudes, practices, and diet quality (Melesse & van den Berg, 2021). In Ghana, people were generally aware of iron deficiency anemia and its association with poor eating habits (Awuah et al., 2021; Rose Omari, 2017). Therefore, informing people of the potential benefits of *akokono* consumption on their iron status could serve in increasing their willingness to consume and purchase *akokono* and *akokono*‐based products. Consumer knowledge could also be associated with awareness of the product's existence and availability (Owureku‐Asare et al., 2016), thus underlining the importance of informing Ghanaians about the existence of *akokono* and *akokono*‐based products and their access points.

Finally, some participants mentioned being motivated to accept *akokono* because it was produced locally which would decrease unemployment. A study conducted by Adams and colleagues concluded that the domestication of palm weevil larvae was financially viable at the micro‐scale and could have practical implications for small‐scale enterprise development in addressing problems of malnutrition and unemployment (Adams et al., 2021).

## 5 CONCLUSION

This study found that the addition of palm weevil larvae improved the nutritional composition of tomato paste, specifically in terms of its protein, fat, zinc, and iron contents. In addition, participants preferred the 15% palm weevil larvae‐fortified tomato paste sample owing mainly to its color and taste. For food‐to‐food fortification to be successful, it is important to recognize the importance of a food's sensory aspects and develop food products with familiar tastes and textures. Health and nutrition were additional common motives for participants' acceptance and willingness to purchase palm weevil larvae and its derived products. We found among participants, however, a lack of knowledge and awareness about palm weevil larva's nutritional benefits, cooking and preparation, income generation potential, and access points. This highlights the need for nutrition education to support the consumption of palm weevil larvae–tomato paste as a potential feasible strategy to curtail micronutrient deficiencies, such as iron deficiency anemia.

Based on the results of our study, we consider that further research should: 1) assess the effect of heat on the nutritional composition of palm weevil larvae‐fortified tomato paste, 2) examine the microbial quality of palm weevil larvae‐fortified tomato paste, and 3) evaluate the impact of daily consumption of palm weevil larvae‐fortified tomato paste on women's iron status.

## AUTHOR CONTRIBUTIONS

**Loloah Chamoun:** Conceptualization (lead); formal analysis (lead); methodology (lead); resources (lead). **Salwa Karboune:** Methodology (equal); supervision (supporting); validation (lead). **Hugo Melgar‐Quinonez:** Supervision (supporting); writing – review and editing (supporting). **Herman Lutterodt:** Resources (equal); writing – review and editing (supporting).

## CONFLICT OF INTEREST STATEMENT

The authors declare that they do not have any conflict of interest.

## ETHICAL STATEMENTS

This study was approved by the Institutional Review Board of McGill University and Kwame Nkrumah University of Science and Technology.

## INFORMED CONSENT

Written informed consent was obtained from all study participants.

## Supporting information


Supplementary Figures

## Data Availability

The data that support the findings of this study are available on request from the corresponding author. The data are not publicly available due to privacy or ethical restrictions.
