# Supplementary material for: Nutritional composition and consumer acceptance of tomato paste fortified with palm weevil larvae (Rhynchophorus phoenicis Fabricius) in the Ashanti region, Ghana
Source: Food Sci Nutr. 2023 Jul 17;11(8):4583–95. doi: 10.1002/fsn3.3418 (PMC10420775; doi:10.1002/fsn3.3418)
Supplement: Supplementary file 1 — Supplementary Figures [file FSN3-11-4583-s001.pptx]

## Slide 1
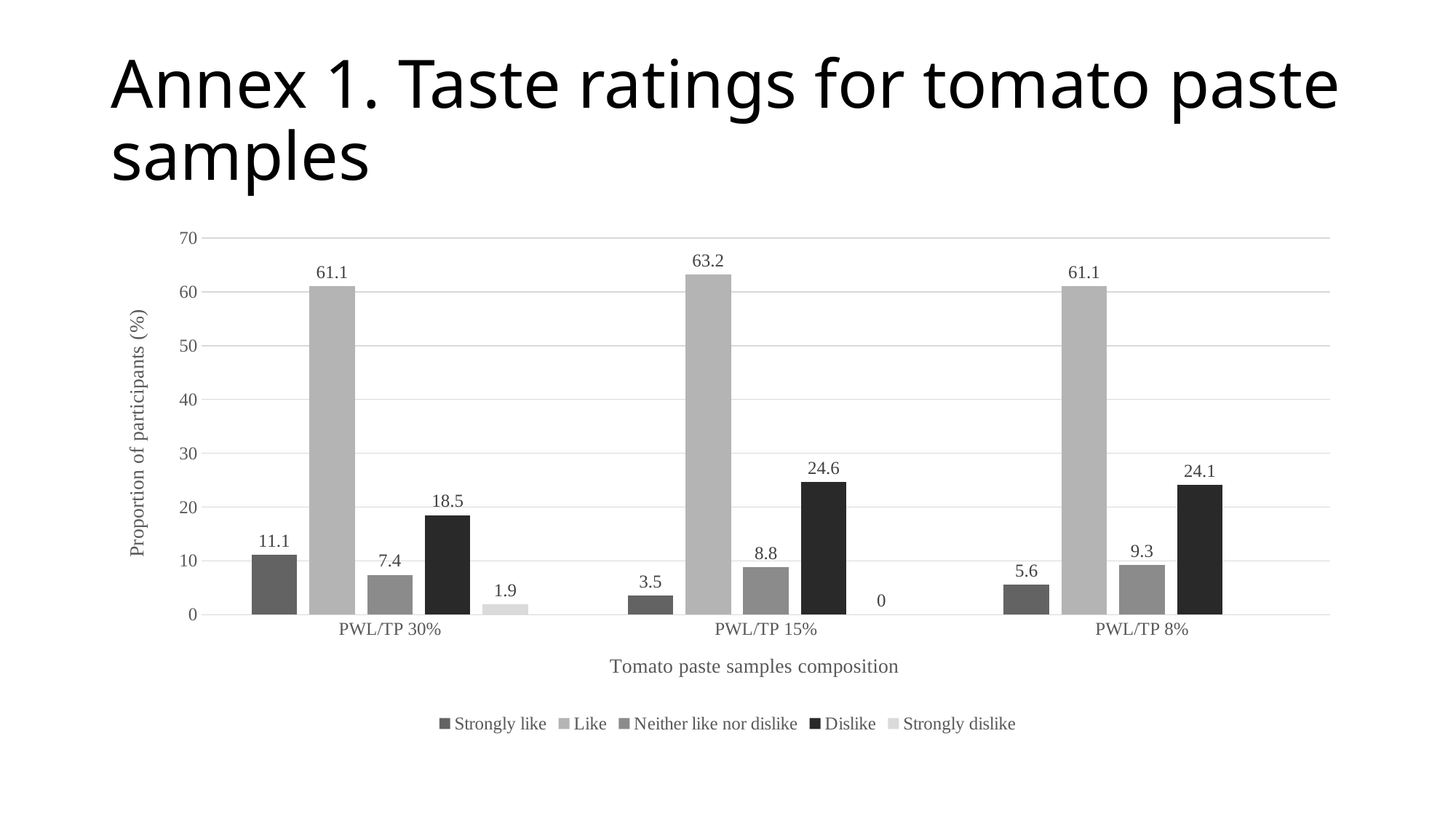

# Annex 1. Taste ratings for tomato paste samples
### Chart
| Category | Strongly like | Like | Neither like nor dislike | Dislike | Strongly dislike |
|---|---|---|---|---|---|
| PWL/TP 30% | 11.1 | 61.1 | 7.4 | 18.5 | 1.9 |
| PWL/TP 15% | 3.5 | 63.2 | 8.8 | 24.6 | 0.0 |
| PWL/TP 8% | 5.6 | 61.1 | 9.3 | 24.1 | None |

## Slide 2
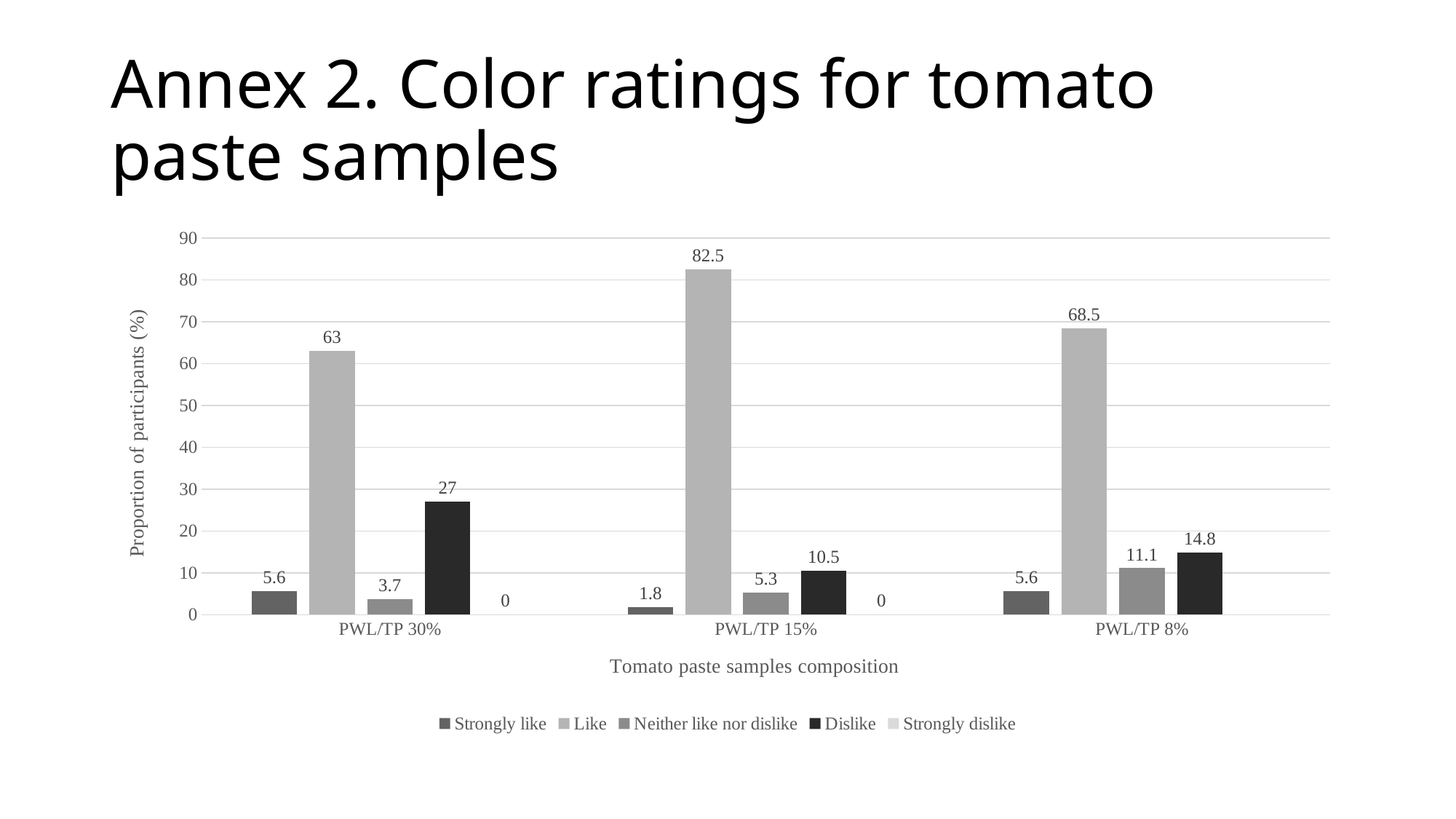

# Annex 2. Color ratings for tomato paste samples
### Chart
| Category | Strongly like | Like | Neither like nor dislike | Dislike | Strongly dislike |
|---|---|---|---|---|---|
| PWL/TP 30% | 5.6 | 63.0 | 3.7 | 27.0 | 0.0 |
| PWL/TP 15% | 1.8 | 82.5 | 5.3 | 10.5 | 0.0 |
| PWL/TP 8% | 5.6 | 68.5 | 11.1 | 14.8 | None |

## Slide 3
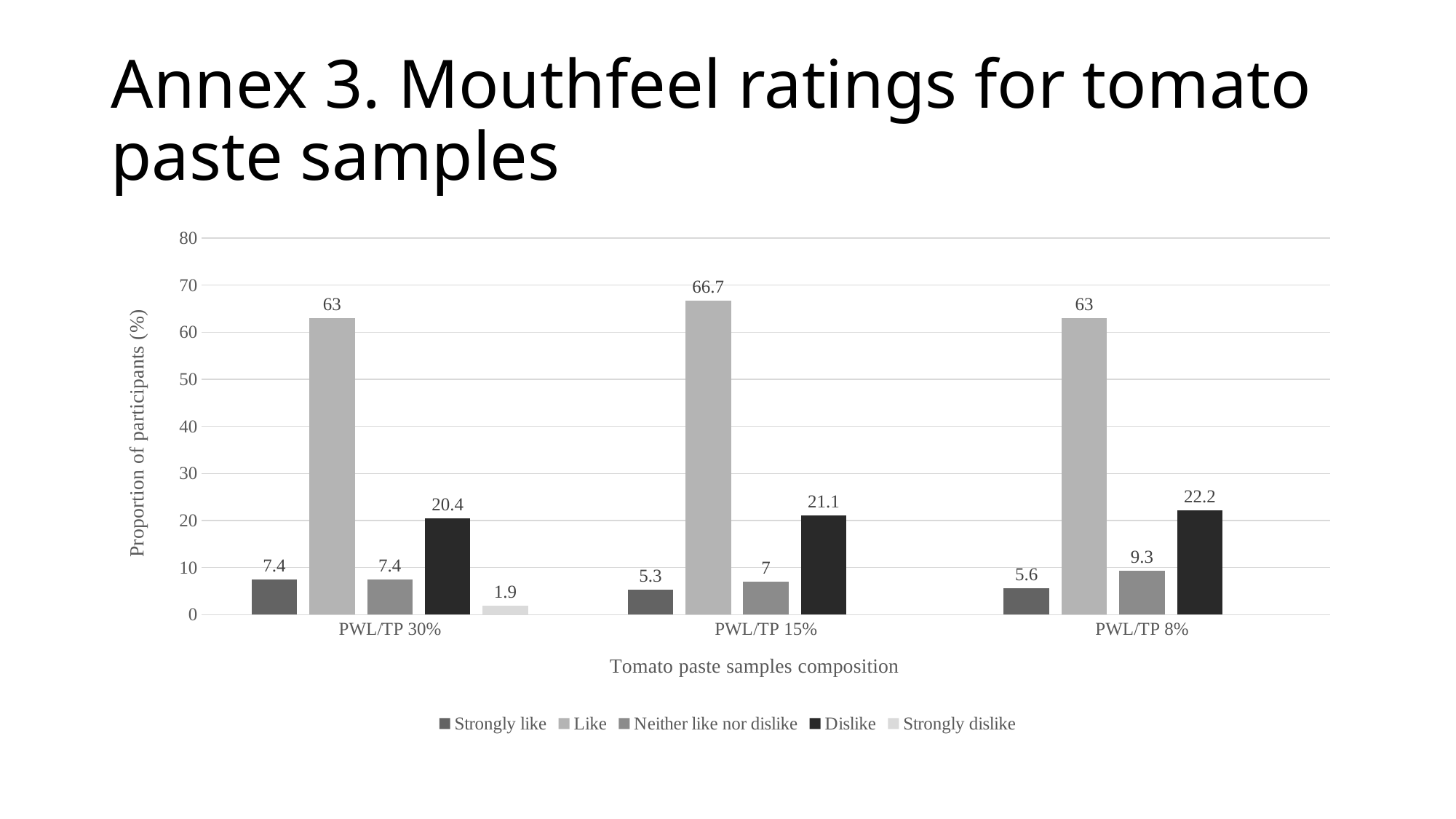

# Annex 3. Mouthfeel ratings for tomato paste samples
### Chart
| Category | Strongly like | Like | Neither like nor dislike | Dislike | Strongly dislike |
|---|---|---|---|---|---|
| PWL/TP 30% | 7.4 | 63.0 | 7.4 | 20.4 | 1.9 |
| PWL/TP 15% | 5.3 | 66.7 | 7.0 | 21.1 | None |
| PWL/TP 8% | 5.6 | 63.0 | 9.3 | 22.2 | None |

## Slide 4
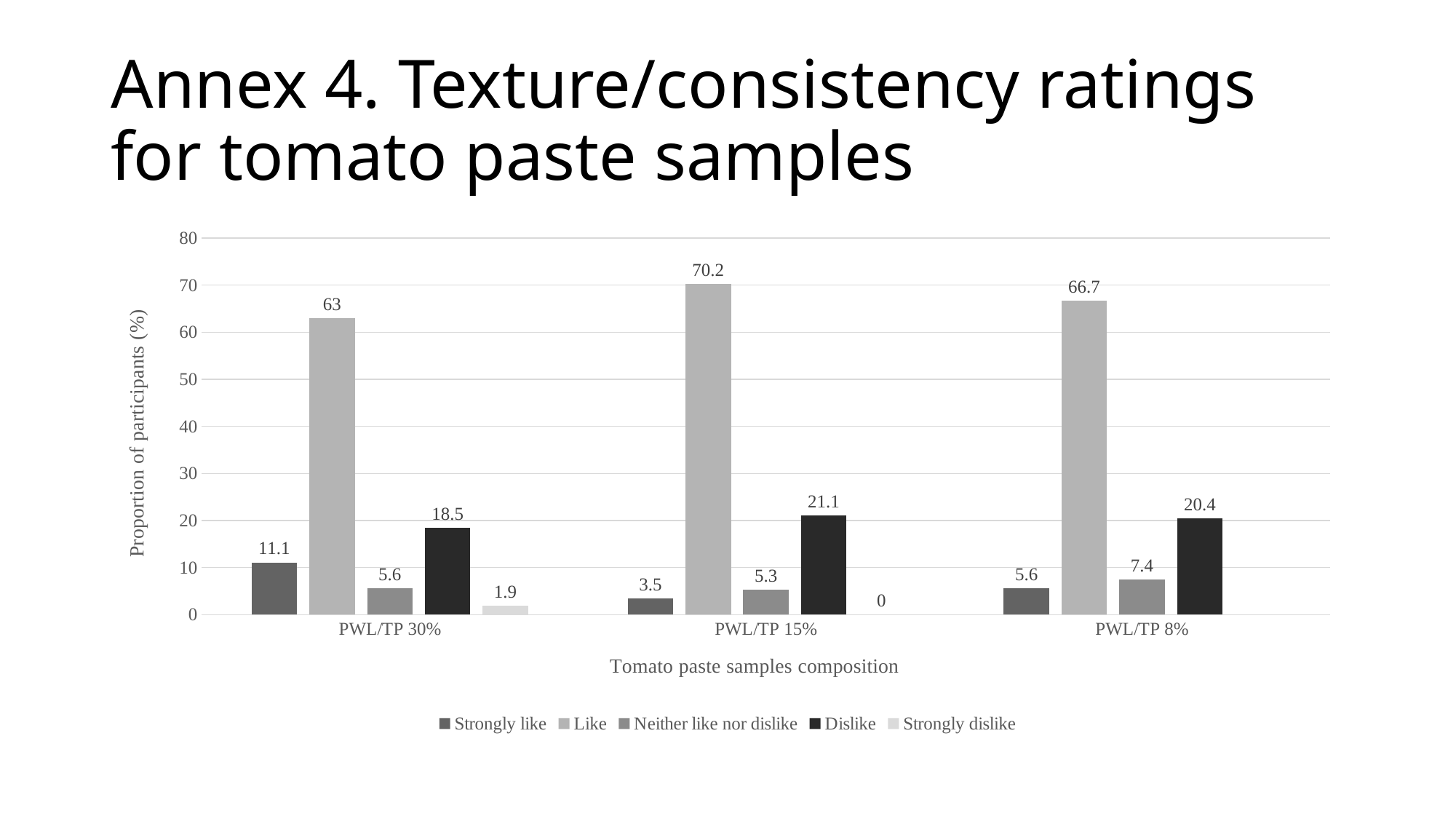

# Annex 4. Texture/consistency ratings for tomato paste samples
### Chart
| Category | Strongly like | Like | Neither like nor dislike | Dislike | Strongly dislike |
|---|---|---|---|---|---|
| PWL/TP 30% | 11.1 | 63.0 | 5.6 | 18.5 | 1.9 |
| PWL/TP 15% | 3.5 | 70.2 | 5.3 | 21.1 | 0.0 |
| PWL/TP 8% | 5.6 | 66.7 | 7.4 | 20.4 | None |
